# A six-generation Chinese family in haplogroup B4C1C exhibits high penetrance of 1555A > G-induced hearing Loss

**DOI:** 10.1186/1471-2350-11-129

**Published:** 2010-09-07

**Authors:** Yan Bai, Zhengmin Wang, Wenjia Dai, Qingzhong Li, Guoling Chen, Ning Cong, Minxin Guan, Huawei Li

**Affiliations:** 1 Department of Otolaryngology, Eye & ENT Hospital, Fudan University, 83 Fenyang Road, Shanghai, 200031, China; 2 Department of Otolaryngology, Affiliated Chongqing Children's Hospital, Chongqing Medical University, 136 Zhongshan Er Road, Chongqing, 400014, China; 3 Department of Otolaryngology, the First Affiliated Hospital, Wenzhou Medical College, Wenzhou, Zhejiang, China; 4 Institute of Biomedical Sciences, Fudan University, Shanghai, 20031, China

## Abstract

**Background:**

The 1555A > G mutation is the most common cause of aminoglycoside-induced and non-syndromic deafness. However, the variable clinical phenotype and incomplete penetrance of A1555G-induced hearing loss complicate our understanding of this mutation. Environmental factors, nuclear genes, mitochondrial haplotypes/variants and a possible threshold effect have been reported to may be involved in its manifestation.

**Methods:**

Here, we performed a clinical, molecular, genetic and phylogenic analysis in a six-generation Chinese family.

**Results:**

A clinical evaluation revealed that affected individuals without aminoglycoside exposure developed hearing loss extending gradually from 12000 Hz to 8000 Hz and then to 4000 Hz. Using pyrosequencing, we detected an identical homoplasmic 1555A > G mutation in all individuals except one. We did not find any correlation between the mutation load and the severity of hearing loss. T123N coexisted with the 1555A > G mutation in six affected subjects in our pedigree. Analysis of the complete mtDNA genome of this family revealed that this family belonged to haplotype B4C1C and exhibited high penetrance. Upon the inclusion of subjects that had been exposed to aminoglycosides, the penetrance of the hearing loss was 63.6%.; without exposure to aminoglycosides, it was 51.5%. This pedigree and another reported Chinese pedigree share the same haplotype (B4C1C) and lack functionally significant mitochondrial tRNA variants, but nevertheless they exhibit a different penetrance of hearing loss.

**Conclusions:**

Our results imply that the factors responsible for the higher penetrance and variable expression of the deafness associated with the 1555A > G mutation in this pedigree may not be mtDNA haplotype/variants, but rather nuclear genes and/or aminoglycosides.

## Background

A number of mitochondrial mutations have been described to be associated with non-syndromic and syndromic hearing loss. The 1555A > G mutation is the most common mutation attributed to aminoglycoside-induced and non-syndromic deafness. It was first described in a large Arab-Israeli family that exhibited maternally inherited non-syndromic deafness [1].

The first family with aminoglycoside-induced hearing impairment was reported by Higashi K [2]. Aminoglycosides exert their antibacterial effect by specifically binding to the bacterial ribosome, thus inhibiting protein synthesis or inducing mistranslation of messenger RNAs [3]. In 1993 Prezant et al. [1] first determined that the 1555A > G mutation causes the structure of the mitochondrial 12sRNA to be more similar to that of bacterial rRNA, thus rendering the mitochondrial ribosomal decoding site more available to aminoglycoside antibiotics. This strengthens the binding of aminoglycosides to mitochondrial RNA and causes hypersensitivity to aminoglycoside ototoxicity [4-6]. Clinical phenotypes due to 1555A > G mutation may vary among maternal relatives within families or among different families, ranging from severe pre-lingual hearing loss to moderate progressive hearing loss of later onset to no hearing loss at all in some individuals. However, many studies have suggested that the 1555A > G mutation leads to only mild dysfunction and sensitivity to aminoglycosides, as it alone was insufficient to produce the variable phenotypes of deafness. In contrast, patients carrying the 1555A > G mutation can also suffer from hearing loss without any aminoglycoside exposure [1,7,8]. Thus the genotype-phenotype relationship between the 1555A > G mutation and deafness is very complicated.

Phenotypic heterogeneity is an unsolved problem of mitochondrial disorders. Owing to the presence of heteroplasmy, the proportion of mutant mtDNA might be one of the causes of the variable penetrance and severity of mitochondrial disorders. There may be a threshold effect for both the clinical expression and biochemical defects [8]. In addition, many modifiers are suspected to be involved, including environmental factors (e.g. aminoglycoside antibiotics), nuclear genes and mitochondrial haplotype/variants [9,10]. Mutations in the nuclear genes GJB2 and TRMU are reported to influence the degree of hearing loss in patients with the 1555A > G mutation [11,12]. Recently, many mitochondrial tRNA variants have been reported to modulate the penetrance of the 1555A > G mutation [12-14]. However, the mechanisms of the phenotype variation and the incomplete penetrance of hearing loss in subjects harboring the 1555A > G mutation have remained unclear.

Here, we investigated a six-generation Han Chinese family with aminoglycoside-induced and non-syndromic hearing loss. An identical 1555A > G mutation was found in all members of this pedigree. We then performed an evaluation of the possible modifiers mentioned above by 1) quantification of the 1555A > G mutation load by pyrosequencing (Biotage AB, PSQ96MA) and 2) analysis of mutations in the nuclear gene GJB2 and TRMU and 3) phylogenetic tree analysis to identify the mitochondrial haplogroup.

## Methods

### Participants and audiologic evaluation

**Figure 1:**
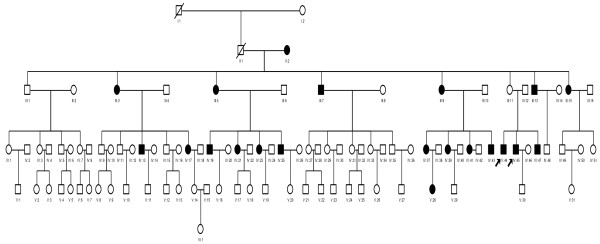
**Pedigree of a six-generation Chinese Han family that harbors the 1555A > G mutation with aminoglycoside-induced and nonsyndronic hearing loss**. Individuals with hearing loss are indicated by filled symbols. The arrow indicates the proband. All matrilineal relatives of this family were homoplasmic for the 1555A > G mutation except for subject II2 who was heteroplasmic.

This study was conducted in accordance with the policies of the Ethics Committee of Eye & ENT Hospital, Fudan University. A six-generation Han Chinese family with 103 members is shown in Figure 1. Thirty three of them were examined at the Eye & ENT Hospital of Fudan University after written informed consent was obtained from all participants. A comprehensive history interview was conducted with all participants and a detailed physical examination by professional otolaryngologists was performed to ensure that there were no other abnormalities except for deafness. Appropriate audiological examinations were carried out, including otoscopic examination, pure-tone audiometry (PTA), otoacoustic emissions, tympanometry, acoustic reflexes, auditory brainstem response (ABR). PTA was calculated from the average of audiometric thresholds at 500, 1000, 2000, 4000 and 8000 Hz. The severity of hearing impairment was classified as follows: normal < 26 decibel (dB) hearing level (HL); mild: 26-40 dBHL; moderate: 41-70 dBHL; severe: 71-90 dBHL; profound: > 90 dBHL.

### mtDNA genome analysis

Genomic DNA was extracted from peripheral blood using a Paxgene blood DNA kit (Qiagen, Hongkong, Germany). The entire mitochondrial genomes of the two probands(IV44, IV45) and one maternal member in this pedigree were amplified by polymerase chain reaction (PCR) in 24 overlapping fragments as described elsewhere[15]. The PCR products were purified and subsequently analyzed by direct sequencing analysis in an ABI 3700 automated DNA sequencer. Sequences were compared with the revised Cambridge reference sequence (rCRS) of the human mitochondrial DNA (Accession No. AC_000021. 2). Oligodeoxynucleotides corresponding to 12SrRNA and tRNA were also used.

### Quantification of the mtDNA 1555A > G mutation load

DNAs samples from an unrelated person with normal hearing (PTA <26 dB) and a person identified as having the 1555A > G mutation were amplified using the following pair of primers: Forward 5'-CGATCAACCTCACCACCTCT-3' and Reverse 5'-TGGACAACCAGCTATCACCA-3'. PCR products were cloned into a pGEM-T vector and plasmids of all colonies were sequenced. Two clones were identified, one with the wild-type genotype and the other with the 1555A > G mutation. The wild-type and mutated DNA were then mixed to generate samples with the 1555A > G mutation present at gradient levels ranging from 0%-100%. Specific SNP assays were performed by pyrosequencing with the following primers: Forward 5'-ACATTTAACTAAAACCCCTACGCA-3', Reverse 5'-AGTTGGGTGCTTTGTGTTAAGCT-3' and Sequencing 5'-CACTTACCATGTTACGACT-3'. Each sample was analyzed in triplicate by pyrosequencing assays to determine the detection threshold. Sequencing identification was performed automatically by the SQA software.

### Mutational analysis of the GJB2 gene

Mutations in the GJB2 nuclear gene were analyzed. The primers used for screening the entire coding region of GJB2 mutation have been described elsewhere (Forward 5'-TTGGTGTTTGCTCAGGAAGA-3' Reverse 5'-GGCCTACAGGGGTTTCAAAT-3') [16]. PCR products were sequenced and compared with the wild-type GJB2 sequence (Accession No. NM_004004.5).

### Mutational analysis of the TRMU gene

The forward and reverse primers for the A10 S variant in the TRMU gene are 5'-ACAGCGCAGAAGAAGAGCAGT-3' and 5'-ACAACGCCACGACGGACG-3', repectively. The PCR segments were subsequently analyzed and compared with the TRMU genomic sequence (Accession No. AF_448221) [17].

### Analysis of mitochondrial haplotype and statistical analysis

Phylogenetic trees were used to analyze the haplogroups, including mtDB Http://www.genpat.uu.se/mtDB and the recently updated East Asian mtDNA phylogeny [18]. A logistic procedure and odds ratio estimate were used to assess the correlation between age and deafness. The analysis of covariance was used to assess the factors. The hearing thresholds per frequency was used as dependent variable, the presence of 1555A < G mutation, T123N variant in GJB2 gene, age, aminoglycoside exposure as covariates.

## Results

### Clinical features and auditory findings

**Figure 2:**
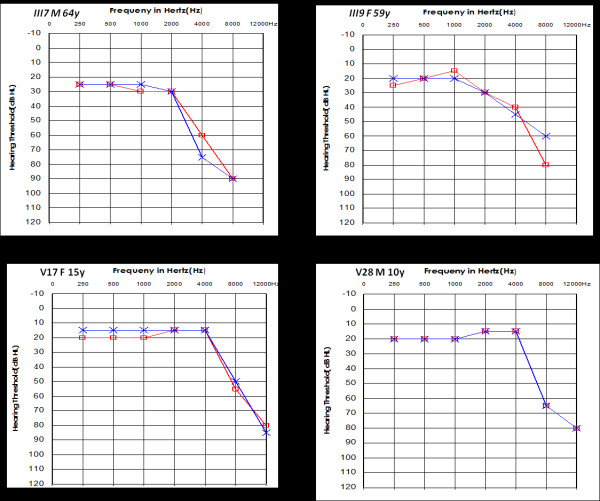
**Audiograms of some affected subjects**.

All patients came from a large Han Chinese family living in Zhejiang Province (Figure 1). Computed tomography scans of the temporal bones of the two probands (IV44, IV45) ruled out congenital ear malformations. Hearing loss was the only symptom and was ascertained at the Eye & ENT Hospital of Fudan University. The pattern of inheritance was typical maternal transmission. After a comprehensive clinical review and audiological and molecular biological examination, we found that 21 out of 33 maternal members carrying the 1555A > G mutation exhibited bilateral and symmetrical sensorineural hearing loss, which was especially severe at high frequencies. Four of the affected matrilineal relatives in the fourth generation had a history of aminoglycoside injection. Subjects V-17 and V28, who displayed normal hearing and mild deafness, respectively, shared similar audiograms. There was a slope of hearing loss at high frequency (8000 Hz) and more severe loss at extended high frequency (12000 Hz), while the hearing at frequencies 500 Hz, 1000 Hz, 2000 Hz and 4000 Hz was normal. In the third generation, III7 and III9 showed mild-to-moderate hearing loss, with normal hearing at 500 Hz, 1000 Hz and 2000 Hz, but an obvious loss at high frequencies (4000 Hz, 8000 Hz) and no response at 12000 Hz (Figure 2). The exact timing of aminoglycoside exposure, audiometric configuration, severity of deafness and age of onset are shown in Table 1. The penetrance of hearing loss in this pedigree was 63.6% (included aminoglycosides received) and 51.5% (excluded aminoglycosides received).

**Table 1:** Clinical analysis, molecular evaluation and 1555A > G quantification in a six-generation pedigree with hearing loss

	Age(year)	PTA(dB) right ear	PTA(dB) Left ear	Audiometric configuration	Severity of hearing loss	GJB2	A1555G (A%)	A1555G (G%)	Use of aminoglycosideand age of onset(years)
II2	91	86	73	sloping	severe		33.4	66.6	
III3	56	27	31	sloping	mild		4.4	95.6	
III5	66	93	57	sloping	moderate		0	100	
III7	64	43	49	sloping	moderate	T123N	0	100	
III9	59	37	35	sloping	mild		4.1	95.9	
III11	56	26	23		normal		0	100	
III13	54	30	26	sloping	mild		0	100	
III15	48	26	26	sloping	mild		0	100	
IV13	45	100	103	sloping	profound		0	100	
IV17	49	95	88	sloping	severe		0	100	18 years streptomycin
IV19	42	85	71	sloping	severe		3.4	96.9	
IV21	39	79	66	sloping	moderate	T123N	5.1	94.9	
IV23	35	82	85	sloping	severe	T123N	0	100	Congenital gentamycin
IV25	33	103	101	sloping	profound		0	100	4 years streptomycin
IV37	34	81	81	sloping	severe		2.2	97.8	7-8 months uncertain
IV39	32	98	98	sloping	profound	V27I T123N	0	100	5 years fever streptomycin
IV41	28	> 95	> 95	sloping	profound		0	100	
IV43	26	103	100	sloping	profound	V27I T123N	0	100	3 years fever uncertain
IV45	34	98	98	sloping	profound	I203T	2.3	97.7	Congenital uncertain
IV47	32	88	90	sloping	severe	I203T	0	100	4 years uncertain
V28	10	27	27	sloping	mild	T123N	0	100	
V17	15	25	24		normal	V27I	0	100	
V18	6	25	22		normal		2.7	97.3	
IV49	26	23	23		normal	V27I T123N	0	100	

### Mitochondrial DNA and haplogroup analysis

**Figure 3:**
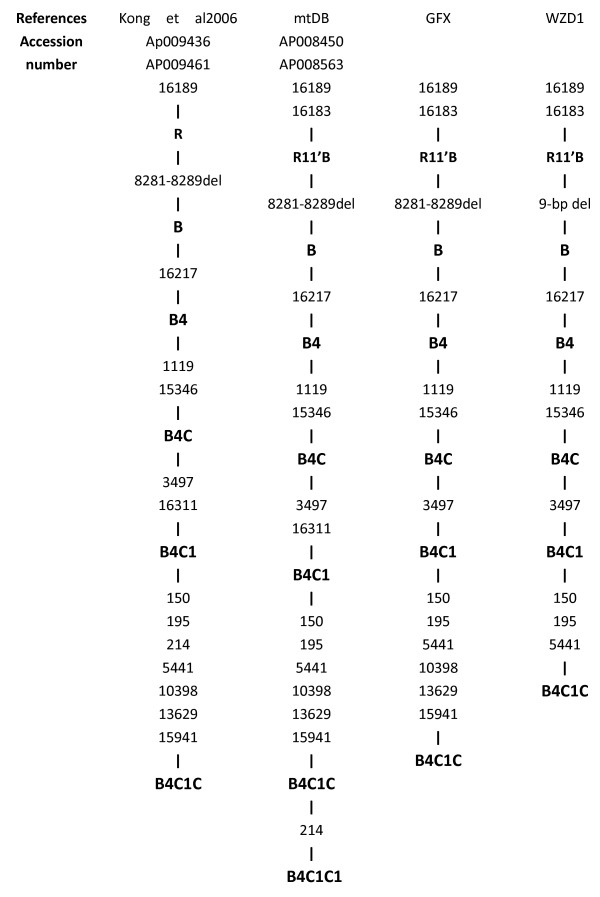
**Classification tree of the two pedigrees using the Updated East Asian mtDNA phylogenetic tree and mtDB **Http://www.genpat.uu.se/mtDB.

We sequenced the mitochondrial 12SrRNA and tRNA genes of all participants in this pedigree (GFX). By direct sequencing, the 1555A > G mutation was the only detected pathogenic mutation. To assess the association of the mitochondrial haplotype with the penetrance and phenotypic expression of the 1555A > G mutation, the two probands (IV44, IV45) and another affected relative received a complete mitochondrial genomes spanning and sequencing. Many variants were detected (Table 2). According to the recently updated East Asian mtDNA phylogeny and mtDB, this pedigree thus can be grouped into haplotype B4C1C (Figure 3) [14].

**Table 2:** mtDNA variants in two Chinese pedigrees, GFX and WZD1

Gene	position	replacement	Conservation	GFX	**WZD1**^**b**^	**Previously reported**^**c**^
			
			**(H/B/M/X)**^**a**^			
D-loop	73	A >G		G	G	yes
	150	C > T		T	T	yes
	195	T > C		C	C	yes
	263	A > G		G	G	yes
	310	T > CTC			CTC	yes
	316	InsetC		insetC		no
	392	T > C		C		no
	16140	T > C			C	Yes
	16182	A > C			C	Yes
	16183	A > C		C	C	Yes
	16189	T > C		C	C	Yes
	16217	T > C		C	C	Yes
	16274	G > A			A	Yes
	16305	A > T			T	Yes
	16335	A > G			G	Yes
	16362	T > C		C		Yes
	16519	T > C		C		yes
	16545	T > C		C		no
12SrRNA	750	A > G	A/A/A/>	G		yes
	1119	T > C	T/T/T/C	C	C	Yes
	1438	A > G	A/A/A/G	G	G	Yes
	1555	A > G	A/A/A/A	G	G	Yes
16SrRNA	2706	A > G	A/G/A/A	G	G	Yes
	3107	delC			delC	Yes
ND1	3497	C > T	A/A/L/S	T	T	Yes
	3571	C > T	L/L/L/L		T	Yes
	3738	C > T			T	Yes
ND2	4769	A > G		G	G	Yes
	5441	A > G		G		Yes
CO1	7028	C > T		T	T	Yes
CO2	8200	T > C			C	Yes
	8257	A > G			G	Yes
NC7	8271 > 79	9 > bpdel			9 > bpdel	Yes
	8281 > 89	9 > bpdel		9 > bpdel		No
ATP6	8860	A > G(Thr to Ala)	T/A/A/T	G	G	Yes
CO3	9827	C > T		T		Yes
ND3	10398	A > G(Thr to Ala)	T/T/T/A	G		Yes
ND4	11719	G > A		A	A	Yes
ND5	13629	A > G		G		No
CYB	14766	C > T		T		Yes
	15301	G > A		A		Yes
	15326	A > G(Thr to Ala)	T/M/I/I	G	G	Yes
	15346	G > A(His to Asp)	H/H/H/H	A	A	Yes
tRNA^Thr^	15941	T > C		C		Yes

A Conservation of variant in human(H), bovine(B), mouse(M) and xenopus laevis(X)

b X.Tang et al(2007)[14]

c http://www.mitomap.organdmtBD

### Mutational analysis of the nuclear gene GJB2 and TRMU

To explore the role of the GJB2 gene and the A10 S variant in the TRMU gene in the hearing loss phenotype of this pedigree, we screened GJB2 and A10 S variant in maternal individuals. The V27I (79G > A) and I203T (608T > C) in the GJB2 gene were detected as homozygous status in maternal relatives. Seven maternal relatives, six of whom suffered from hearing loss were found to be heterozygous for the T123N (368T > C) SNP (Table 1). We failed to detect any variant in TRMU in affected maternal relatives who did not have a history of exposure to aminoglycosides.

### Detection of the threshold of the 1555A > G mutation

**Figure 4:**
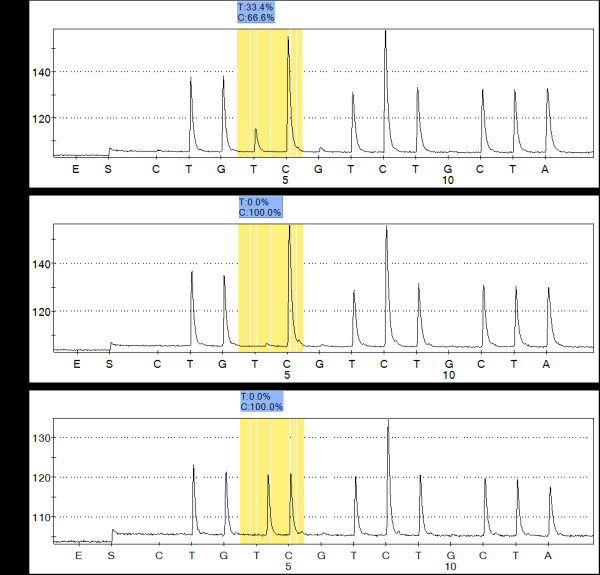
**Quantification of mtDNA 1555A > G mutation load by pyrosequencing**. A Sample II2 heteroplasmic for A1555G. B Sample homoplasmic for A1555G. C Sample for wild-type genotype. Select 3 Pyrograms for representative samples. The boxes show the AQ values obtained for the allele. The value for the mutated allele represents the level of heteroplasmy in the samples.

Using pyrosequencing, we examined all maternal relatives, one spouse in this pedigree and one unrelated individual. All matrilineal relatives of this family were found to be homoplasmic for the 1555A > G mutation except for subject II2 who was heteroplasmic. The exact load of the mutation in each subject is shown (Table 1, Figure 4).

## Discussion

The hereditary pattern of aminoglycoside hypersensitivity is usually consistent with maternal transmission, implying mitochondrial genome involvement. In our study, we found that in six generations of a Han family affected with aminoglycoside-induced and non-syndromic hearing loss, the mode of inheritance was strictly maternal. Therefore, we performed a series of tests for mitochondrial disease, including clinical examination, molecular biological investigationl, genetic analysis, pyrosequencing and phylogenetic tree analysis for this pedigree. The mtDNA analysis indicated that the 1555A > G mutation was the only pathogenic mutation. Furthermore, we detected the identical homoplasmic 1555A > G mutation in all members of this pedigree except II2, who was heteroplasmic for the mutation. In addition, the pedigree exhibited a high penetrance and phenotypic expression of hearing loss carrying 1555A > G mutation with 63.6% of the family members suffering from hearing loss.

Pyrosequencing technology is an accurate, sensitive and specific way to detect heteroplasmy and to quantify the mutation load [19]. Pyrosequencing was used by Ballana et al [19] to detect unrecognized low-level mtDNA heteroplasmy in a three-generation heteroplasmic family harboring the 1555A > G mutation. Similar to Castillo, they found a slight correlation between the heteroplasmic mutation load and the severity of hearing loss [19,20]. Interestingly, we did not find such a correlation in the present study.

It has been suggested that mtDNA haplotypes could modulate the penetrance and phenotype of mtDNA mutations. The phylogenetic tree can be a very helpful approach to assessing the origin of an mtDNA mutation [21,22]. Phylogenetic analyses of a family with Leber's hereditary optic neurophy (LHON) suggested that the mtDNA haplotype J increased the penetrance of a disease-causing LHON mutation in Europe [23]. In contrast, Torroni et al [24] concluded that the specific European haplotype had no effect on the clinical phenotype of the mtDNA A3243G mutation. To assess the effect of haplotype on disease penetrance, we performed a complete mitochondrial genomic analysis in this pedigree (GFX). In addition to the identical 1555A > G mutation, we found a set of mtDNA polymorphisms. According to the recently updated East Asian mtDNA phylogenetic tree and mtDB, we were able to classify this pedigree as haplotype B4C1C based on the observed variants (A16183C, T16189C, 9-bpdel, T16217C, T1119C, G15346A, C3497T, C150T, T195C, A5441G, A10398G, A13629G, T15941C) [21]. Interestingly, another Chinese pedigree (WZD1), which is known to be associated with 1555A > G-induced hearing loss, turned out to be haplotype B4C1C rather than haplotype B4C1C1, according to the haplotype system developed by Tanaka et al [25]. This pedigree showed a strikingly low penetrance of hearing loss (5.9%). Thus, with the effect of aminoglycosiders included, the penetrance of hearing loss in these two Chinese families was 63.6% and 5.9%, respectively. Without the effect of aminoglycosides, the penetrance in the two pedigrees was 51.5% and 0%, respectively. Interestingly, the two pedigrees shared the same mitochondrial haplotype (B4C1C) in the same racial background and yet exhibited different penetrances. Thus, the mtDNA haplotype may not have any effect on penetrance. Torroni et al [26] used a phylogenetic tree to analyze 50 Spanish families and 4 Cuban families carrying the 1555A > G mutation, and they found that different mtDNA haplotypes are not responsible for the variable phenotype of the 1555A > G mutation. This finding was further strengthened by Casano et al. in their studies of an Italian family [27]. However, the effect of mtDNA haplotype on penetrance could differ between regional groups, as the mtDNA of European and East Asians occupy different branches of the world phylogeny, In their reappraisal of the complete mtDNA of Chinese and Japanese families suffering from deafness, Yao et al [21]concluded that the mtDNA background may not play a decisive role in modulating the phenotypic expression of the 1555A > G mutation in an East Asian background. While sharing the same ethnic and haplotypic background, both the pedigree in our study and pedigree WZD1 exhibited strikingly different clinical phenotypes and penetrances. This finding as well as those of Yao, Torroni and Casano, indicates that haplotype B4C1C may not play a substantial role in the penetrance and expression of the 1555A > G mutation associated with deafness. Given the mounting evidence that haplotype (whether in different racial groups or in a similar racial background) does not have a major effect on penetrance, there may be other factors. Recently, Guan et al [12-14] reported that mitochondrial tRNA^Glu^A14693, tRNA^Thr^T15908C, tRNA^Arg^T10454C, tRNA^Ser(UCN)^G7444A, tRNA^Cys^T5802C, tRNA^Thr^G15927A, tRNA^Cys^G5821A, ND5T12338C and ND4G11696A seemed to account for the higher penetrance and expressivities in many Chinese pedigrees associated with the 1555A > G mutation and deafness. Conversely, they postulated that the absence of those functionally significant mutations in tRNA and rRNAs or a secondary LHON mutation in these Chinese pedigrees might account for the very low penetrance of hearing loss. However, the pedigree that we studied which did not have a secondary mtDNA mutation or the above functionally significant mutations, nevertheless exhibited high levels of penetrance and phenotype of hearing loss. Thus, the role of the mitochondrial haplotype or functional variants in the penetrance and expression of hearing loss seems to be questionable based on our pedigree. Nuclear genes and aminoglycosides may be responsible for the higher penetrance and more variable clinic phenotypes.

To better understand the role of nuclear genes in phenotype variation, we analyzed GJB2 in this pedigree and identified homozygous V27I (79G > A) and I203T (608T > C) and heterozygous T123N (368C > A). Six of the maternal subjects harboring heterozygous T123N had hearing loss except subject V18 (Tab. 1). T123N was absent in other maternally related family members with the 1555A > G mutation. Subject III7, who carried T123N, had the worst hearing in the third generation without any aminoglycoside exposure. In the fourth generation, we found T123N in five affected subjects, of which one(IV21) had moderate hearing loss without a history of aminoglycoside exposure, while the others four, all of whom had aminoglycoside exposure, exhibited severe or profound deafness. It appears that the coexistence of T123N and aminoglicoside use led to more severe deafness. In the fifth generation, T123N was found in subject V18, who had a normal hearing. As V18 was only 6 years old and son of IV21, we performed a logistic procedure and odds ratio estimate to assess the correlation between age and deafness. We found age to be a high risk factor in this pedigree (OR = 1.206). It will be interesting to find out whether IV18 dose experience hearing lose in the future. Based on results above, there may be a slight correlation between hearing loss and T123N (except in the case V18). T123N is located in CL functional domain of GJB2. This mutation was reported to be an unknown disease-causing mutation in the Connexin-deafness mutations database at http://davinci.crg.es/deafness[28] and it was identified in Japanese and Chinese patients. Nevertheless, T123N is not highly conserved at position 123 and this mutation was found in both the control group and the patient group. In addition, was counted as a mutation in a Japanese group but as a neutral variant in a Chinese study [16,29]. Overall T123N was an unclassified mutation in GJB2. Due to its unclear clinical implication and coexistence with the 1555A > G mutation in six affected subjects in our pedigree, we suspect that the heterozygous T123N may be relevant of the phenotype expression and penetrance of hearing loss in this pedigree. However, this interpretation should be taken with caution, as determining the exact pathogenic role of T123n will require further investigation and functional tests.

It was reported that the A10 S mutation in TRMU gene modulated the phenotypic expression of the 1555 A> G mutation in the Israeli/European pedigrees [17]. To assess the role of the A10 S variant in this pedigree, we carried out a mutational screening of exon 1 in TRMU gene, We failed to detect any variant in TRMU exon 1 in affected maternal subjects of this pedigree. So, the absence of A10 S variant in gene TRMU ruled out it involvement in the phenotypic expression of the 1555 A> G mutation in this Chinese family.

In our clinical evaluation, we noticed that the degree of the deafness in fourth generation was worse than in the third and fifth generations. Almost of all the affected subjects in the fourth generation presented severe or profound hearing loss with a clear history of aminoglycoside exposure or uncertain history of aminoglycoside, while those in the fourth and fifth generations without histories of aminoglycoside exposure showed normal or mild hearing loss. As II-2 was a 102-year-old woman with severe hearing loss, her hearing loss might be a consequence of age-related hearing impairment combined with the specific genetic defect. Meanwhile, we noted that IV-25 began suffering hearing loss after only two streptomycin injections at age 4. This is consistent with previous findings that Chinese patients with the 1555A > G mutation suffer severe hearing loss with immediate onset after exposure to a very small dose of aminoglycosides. In contrast, in American patients, the hearing loss is mild and progressive over many years after aminoglycoside administration [10]. Some studies have implicated the cochlear neuroepithelia as the major vulnerable site to aminoglycoside ototoxicity. In general, irreversible cochlear damage is first seen in the outer hair cells of the cochlear basal turn and then in the upper cochlear turns and inner hair cells [30]. The outer hair cells in the basal turn are reported to contain more mitochondria, which suggest that the ability to discern high frequencies is more easily affected than that of low frequencies [31]. Because hearing loss in the affected individual without aminoglycoside exposure is too mild to be discerned by other family members, audiometry is required to diagnose high-frequency hearing loss in these affected relatives. We used high-frequency audiometry to test the affected subjects with mild-moderate hearing loss at extended high frequency (12000 Hz). After analyzing the audiograms of the affected subjects without a history of aminoglycoside exposure, we found the hearing loss of these subjects gradually extended from 12000 Hz to 8000 Hz and then to 4000 Hz (Figure. 2). All subjects were not aware of their hearing loss. Using the high-frequency audiometry, we could detect hearing loss at the 12000 Hz frequency early, particularly in hypersensitive subjects harboring the 1555A > G mutation without histories of aminoglycoside exposure or clinical syndrome. As hearing loss due to the 1555A > G mutation is permanent, early identification could help hypersensitive subjects avoid using amiloglycosides, thus reducing the risk of irreversible cochlear damage.

## Conclusions

In summary, we have analyzed possible modifiers associated with hearing loss due to the 1555A > G mutation. First, we found that the load of the 1555A > G mutation is not correlated with phenotype expression or penetrance in this family. Second, our pedigree exhibits a statistically different penetrance from another reported Chinese pedigree, both of which share haplotype B4C1C and lack functionally significant mitochondrial tRNA or rRNA variants. Our data further strengthen the idea that haplotype background and functional variants may not play a substantial role in the penetrance and expression of the deafness associated with the 1555A > G mutation. Rather nuclear genes and/or aminoglycoside exposure may be responsible for the higher penetrance and variability of hearing loss with the 1555A > G mutation in our pedigree. Third, the covariance analysis showed that m.1555A > G and aminoglycoside exposure are main influence factors, but no frequency specificity. The m.1555A > G places the individual at risk for hearing loss, but the severity and penetrance of hearing lossis based on the interplay of mutation with other factors such as unknown nuclear genes and/or aminoglycoside exposure.

## Competing interests

The authors declare that they have no competing interests.

## Authors' contributions

ZW, HL, MG conceived of the study, and participated in its design and revised the manuscript. ZW provided information of this pedigree. ZW also offered the help of clinical evaluation and coordination, and helped to draft the manuscript. YB performed the molecular genetic studies, participated in the sequence alignment and drafted the manuscript. NC, GC, carried out epidemiological survey. WD, QL reviewed and interpreted the results. All authors had read and approved the final manuscript.

## Pre-publication history

The pre-publication history for this paper can be accessed here:

http://www.biomedcentral.com/1471-2350/11/129/prepub
